# *Plasmodium falciparum* RUVBL3 protein: a novel DNA modifying enzyme and an interacting partner of essential HAT protein MYST

**DOI:** 10.1038/s41598-018-29137-8

**Published:** 2018-07-19

**Authors:** Utsav Sen, Himani Saxena, Juhi Khurana, Akshaykumar Nayak, Ashish Gupta

**Affiliations:** Department of Life Sciences, Shiv Nadar University, Greater Noida, 201314 India

## Abstract

RUVBLs constitute a conserved group of ATPase proteins that play significant role in a variety of cellular processes including transcriptional regulation, cell cycle and DNA damage repair. Three RUVBL homologues, namely, PfRUVBL1, PfRUVBL2 and PfRUVBL3 have been identified in *P*. *falciparum*, unlike its eukaryotic counterparts, which have two RUVBL proteins (RUVBL1 & RUVBL2). The present study expands our understanding of PfRUVBL3 protein and thereby basic biology of Plasmodium in general. Here, we have shown that parasite PfRUVBL3 is a true homolog of human/yeast RUVBL2 protein. Our result show that PfRUVBL3 constitutively expresses throughout the stages of intra-erythrocytic cycle (IDC) with varied localization. In addition to ATPase and oligomerization activity, we have for the first time shown that PfRUVBL3 possess DNA cleavage activity which interestingly is dependent on its insertion domain. Furthermore, we have also identified RUVBL3 to be an interacting partner of an essential chromatin remodeling protein PfMYST and together they colocalize with H3K9me1 histone in parasitophorous vacuole during the ring stage of IDC suggesting their potential involvement in chromatin remodeling and gene transcription.

## Introduction

The complex multistage life cycle of human malaria parasite, *P*. *falciparum* involves two living hosts, the vector mosquito and the human. Both the survival and pathogenicity of Plasmodium depends, not only on smart evasion of host immune response but also on its ability to modulate gene expression at different developmental stages in a timely and efficient manner^1,2^. Despite decades of research, our current understanding of the mechanisms that this parasite employs to regulate its gene expression at transcriptional, post-transcriptional and epigenetic levels, remains poorly understood. We are beginning to explore the role of epigenetic modifications and chromatin modifiers in Plasmodium and understand how different modifications in the chromatin participate in regulating parasite gene transcription. Structural alterations in chromatin architecture are highly dynamic and is facilitated by the actions of chromatin remodeling proteins. Indeed, massive chromatin remodeling is reported to occur during various stages of intra-erythrocytic developmental cycle (IDC) in the parasite’s chromatin^3,4^. Based on whole genome analysis, a repertoire of chromatin remodeling proteins has been identified to exist in the malaria parasite, however only few of them have been functionally characterized in detail^5^. For instance, PfGCN5 and PfMYST are two very well characterized histone acetyltransferase (HAT) proteins in Plasmodium that acetylates histones and are suggested to be recruited onto the promoters of its target genes to regulate their expression^6,7^.

RUVBLs are important ATPase proteins of AAA+ family of enzymes found to be conserved from yeast to humans and are known to play essential role in variety of cellular processes including transcription regulation, apoptosis, epigenetic regulation and DNA damage repair process^8–10^. Unlike eukaryotes including yeast and humans that encode two RUVBL proteins, bioinformatics searches of the *P*. *falciparum* genome identified three putative homologs of RUVBL proteins in Plasmodium, however none of them have been functionally characterized till date^11^. All the three PfRUVBL proteins contains conserved Walker A and Walker B motif, an insertion domain unique to RUVBL proteins and sensor I and sensor II motifs. Most of the studies on parasite RUVBL proteins have employed computational bioinformatics approaches to predict the structural and functional features of Plasmodium RUVBLs. Besides, biochemical studies performed with the recombinant RUVBL proteins have revealed ambiguous results in context of their helicase and ATPase activities and remains inconclusive in absence of point mutational studies^12,13^.

In the present work, we have functionally characterized *P*. *falciparum* RUVBL3 protein in detail showing it to be a true homolog of yeast RUVBL2 protein. Using biochemical and mutational studies, we have shown that in addition to ATPase and oligomerization activity, PfRUVBL3 protein possess a peculiar DNA cleavage activity that is dependent on its insertion domain. Furthermore, we have also identified PfRUVBL3 to be an interacting partner of an essential chromatin remodeling factor PfMYST that suggest PfRUVBL3 to be a potential partner of the chromatin remodeling complexes in Plasmodium and we speculate their potential involvement in regulation of chromatin remodeling and gene transcription.

## Results

### *In silico* analysis, cloning, expression & purification of recombinant PfRUVBL3 and generation of polyclonal antibody

**Figure 1:**
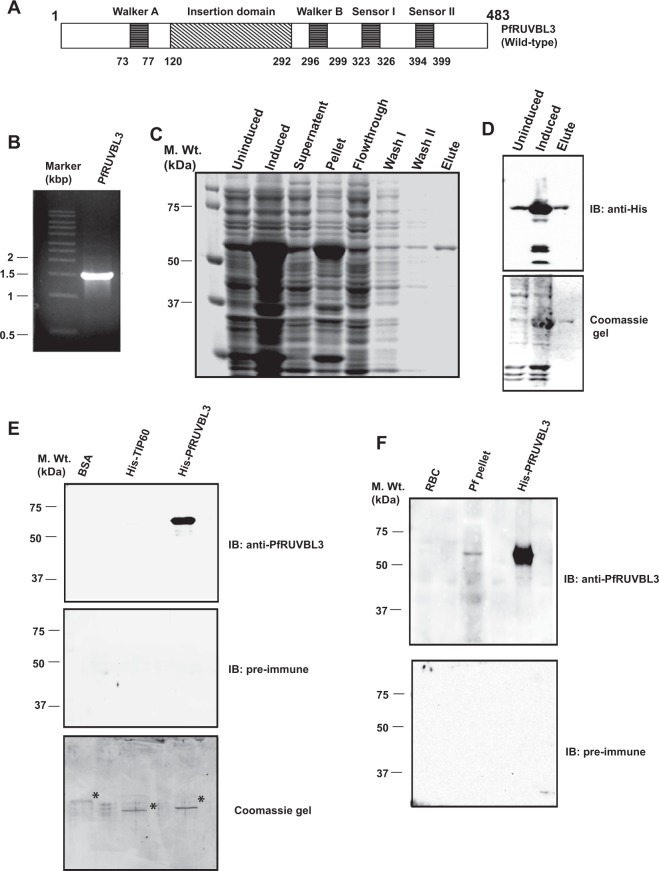
Cloning, expression, purification of PfRUVBL3 protein and generation of anti-PfRUVBL3 polyclonal antibody in rabbit. (**A**) Schematic diagram showing different domain and motifs present in PfRUVBL3 protein (**B**) Agarose gel showing PCR amplification of full-length PfRUVBL3 ORF of ~1.5 kbp (**C**) Coomassie gel showing recombinant protein purification profile of His-PfRUVBL3 protein. BL21 DE3 (codon plus) cells transformed with pET28a-PfRUVBL3 plasmid were cultured till OD reached 0.4–0.6 followed by incubation with 1 mM IPTG for 6–8 hours at 37 °C. Recombinant protein was purified by affinity chromatography using Ni-NTA beads and bound protein was eluted using 500 mM imidazole. (**D**) Western blot of uninduced, induced and purified His-PfRUVBL3 protein by anti-His antibody showed expression and specificity of purified recombinant His-tagged PfRUVBL3 protein. (**E**) Characterization of anti-PfRUVBL3 antibody by Western blot analysis. Purified recombinant proteins were resolved on SDS-PAGE followed by Western blotting using generated anti-PfRUVBL3 antibody or pre-immune. Single band of expected size of ~55 kDa was observed in His-PfRUVBL3 lane only while pre-immune failed to detect any signal under similar conditions. (*) Shows purified protein bands. (**F**) Western blot using anti-PfRUVBL3 antibody to detect endogenous PfRUVBL3 protein. Lysate of mixed stage parasites or uninfected RBCs were resolved on SDS-PAGE followed by Western blotting using anti-PfRUVBL3 antibody or pre-immune sera. Purified His-PfRUVBL3 protein was used as a positive control. Generated PfRUVBL3 antibody detected band of ~55 kDa in parasite lysate and His-PfRUVBL3 lane only while pre-immune sera did not show any cross reaction under similar conditions.

*In silico* homology analysis of all three putative PfRUVBL proteins with *E*. *coli*, *S*. *cerevisiae* and human counterparts revealed that the putative PfRUVBL1 and PfRUVBL2 shows strong homology with human RUVBL1 protein while PfRUVBL3 show homology with human RUVBL2 protein (Supplementary Fig. 1). The domain structure of PfRUVBL3 exhibits the presence of conserved Walker A and Walker B domains, an insertion domain (ID), sensor I and sensor II as depicted in the Fig. 1A.

Full-length PfRUVBL3 clone was generated into pET28a vector followed by purification of recombinant His-PfRUVBL3 protein using affinity chromatography under native condition (Fig. 1B,C). Western blot analysis was performed to detect the expression of His-PfRUVBL3 protein using anti-His antibody and the result showed a band at an expected size of 55 kDa in both the induced bacterial lysate and elute lanes (Fig. 1D).

Polyclonal antibody against full-length PfRUVBL3 was raised by immunization of rabbits with purified protein as per standard protocol for antibody generation. Western blotting was performed to determine the specificity of the newly generated anti-PfRUVBL3 antibody. The result showed that the immunized sera cross-reacted with PfRUVBL3 protein alone while the pre-immune sera failed to detect any signal showing the specificity of generated antibody (Fig. 1E). To examine the ability and specificity of generated antibody to recognize endogenous PfRUVBL3, we performed Western blot analysis with parasite lysate and uninfected RBC lysate was used as a control. Result showed that PfRUVBL3 antibody specifically detected a single band at the expected size of ~55 kDa in parasite-infected RBC lysate only (Fig. 1F). Under similar conditions pre-immune sera did not show any cross-reactivity. Together, these results show that the full-length His-tagged recombinant PfRUVBL3 protein was successfully purified and the generated polyclonal PfRUVBL3 antibody was specific in recognizing both overexpressed and endogenous proteins.

### Putative PfRUVBL3 expresses in all the stages of intra-erythrocytic developmental cycle

**Figure 2:**
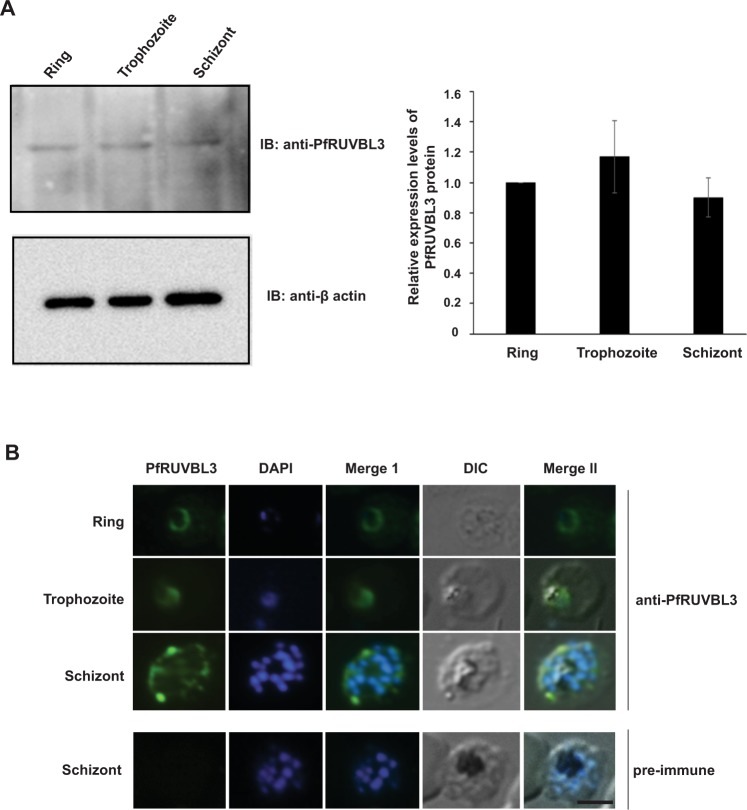
Stage-specific expression and intracellular localization of PfRUVBL3 protein. (**A**) Equal amount of parasite lysate from synchronized culture of ring, trophozoite and schizont stages were resolved on SDS-PAGE followed by Western blotting using anti-PfRUVBL3 or anti-βactin antibody. Band intensities were calculated using Protein Simple machine software and graph was plotted for normalized values taking ring stage value as 1. Full length immunoblots are presented in Supplementary Fig. 6. (**B**) Stage-specific localization of PfRUVBL3 protein. Immunofluorescence assays were performed using anti-PfRUVBL3 antibody or pre-immune on fixed smears of infected erythrocytes harboring various parasite stages of IDC. Green signal depicts localization of PfRUVBL3 protein while blue signal of DAPI stains nucleus. No signal was detected with pre-immune sera under similar experimental conditions. The bar as shown in inset is equivalent to 2 µM.

To examine the expression profile of putative PfRUVBL3 protein during various stages of intra- erythrocytic cycle, equal amount of protein lysate from synchronized parasite cultures of ring, trophozoite and schizont stages (Supplementary Fig. 2) were resolved on SDS-PAGE followed by Western blot analysis with anti-PfRUVBL3 or anti-β actin antibody (Fig. 2A). Band intensities were quantified, and graph was plotted for normalized values. Result showed that PfRUVBL3 protein expresses in all the stages of IDC in contrast to previous report that showed its exclusive expression in schizont stage only^12^. Similarly, subcellular localization of PfRUVBL3 during various stages of IDC was assessed by performing immunofluorescence assay (IFA) using anti-PfRUVBL3 antibody or pre-immune sera. PfRUVBL3 proteins localized in the parasitophorous vacuole of the parasite in the ring stage. However, in trophozoite stage, PfRUVBL3 signal majorly overlapped with DAPI while in schizont stage, it remained predominantly localized at the periphery of infected erythrocyte (Fig. 2B). Together, these results show that PfRUVBL3 protein expresses in all the stages right from ring to schizont however, its localization varies with stage progression of the parasite.

### PfRUVBL3 complements functions of yeast RUVBL2 protein

**Figure 3:**
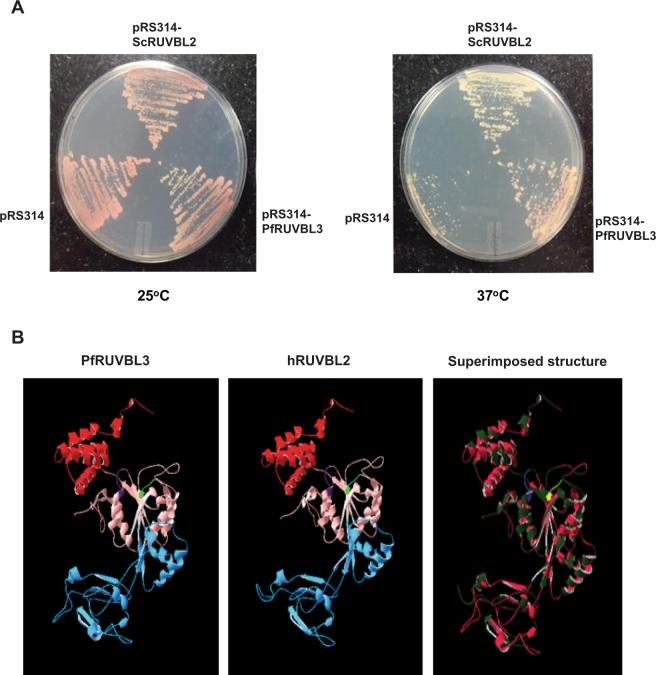
Yeast complementation assay and *in silico* structural analysis of PfRUVBL3. (**A**) *Saccharomyces cerevisiae* temperature-sensitive strain of RUVBL2 (YRVB2a) was transformed with pRS314, pRS314-ScRUVBL2 or pRS314-PfRUVBL3 plasmids and colonies were grown in tryptophan dropout media. Transformed colonies were streaked on to tryptophan-dropout media plate and incubated at 25 °C and 37 °C for 3–5 days. (**B**) Structure of PfRUVBL3 and human RUVBL2 protein. In PfRUVBL3 structure, pink color depicts domain I (1–119 amino acids and 293–359 amino acids), blue color shows domain II (120–292 amino acids), red color shows domain III (360–483 amino acids) and purple & green color depicts Walker A (73–77) and Walker B (296–299) motif respectively. In hRUVBL2 structure, pink color shows domain I (1–126 amino acids and 296–362 amino acids), blue color shows domain II (127–295 amino acids), red color depicts domain III (363–463 amino acids) and purple & green color shows Walker A (80–84) and Walker B (299–302) respectively. Superimposed structure of PfRUVBL3 (pink) and hRUVBL2 (green) shows comparison of both the structures.

Since putative PfRUVBL3 protein showed close sequence similarity with the yeast RUVBL2 we wanted to determine whether PfRUVBL3 is a true homolog of yeast RUVBL2 protein and could functionally complement the function of yeast homolog *in vivo*. To analyze this, we performed complementation assay in yeast using full-length PfRUVBL3 cloned in yeast expression vector pRS314 and yeast RUVBL2 temperature-sensitive (ts) strain. Yeast RUVBL2 ts strain grows normally at permissible temperature of 25 °C however do not survive at restrictive temperature of 37 °C due to degradation of endogenous RUVBL2 protein. We transformed the RUVBL2 ts yeast strain with full-length PfRUVBL3 or ScRUVBL2 or pRS314 vector constructs and were grown at 25 °C and 37 °C. The result showed that the yeast strain transformed either with ScRUVBL2 or PfRUVBL3 plasmids displayed normal growth even at restrictive temperature however, under similar condition, yeast strain transformed with pRS314 empty vector failed to survive (Fig. 3A). At permissive temperature (25 °C), all the transformed yeast cells showed normal growth. Furthermore, to demonstrate that the complementation occurred due to expressed PfRUVBL3 protein, Western blot analysis was performed by taking lysates of pRS314-PfRUVBL3, pRS314-ScRUVBL2 or pRS314 transformed yeast cells using anti-PfRUVBL3 antibody. Result showed the presence of band of expected sizes only in PfRUVBL3 and ScRUVBL2 transformed lanes (Supplementary Fig. 3).

We also generated the structures of PfRUVBL3 and human RUVBL2 protein using Swiss-Model software. Analysis of the superimposed structures showed that PfRUVBL3 protein structure is similar to that of human RUVBL2 protein (Fig. 3B). Together, these results show that PfRUVBL3 protein of *Plasmodium falciparum* is a true homolog of yeast RUVBL2 protein and have structural similarity to its homologs as well.

### PfRUVBL3 shows ATPase, oligomerization and novel DNA cleavage activity

**Figure 4:**
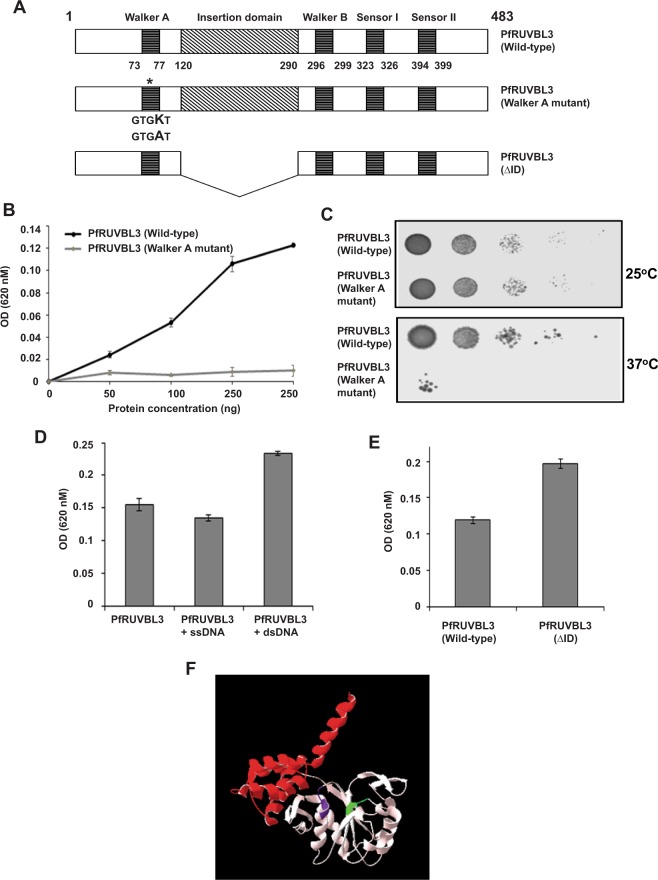
ATPase activity of PfRUVBL3 protein. (**A**) Schematic diagram showing point mutant and deletion mutant of PfRUVBL3 generated for the study. (**B**) ATPase assay was performed using PfRUVBL3 (wild-type) and PfRUVBL3 (Walker A mutant) with indicated concentration and values were obtained at 620 nM OD. Graph was plotted for the average value of three independent experiments performed in duplicate with ±SD. (**C**) Yeast complementation assay with Wild-type and Walker A mutant of PfRUVBL3. ScRUVBL2 temperature-sensitive strain was transformed with pRS314-PfRUVBL3 (Wild-type) or pRS314-PfRUVBL3 (Walker A mutant) and plated on tryptophan dropout media. Serial dilution assay was performed with saturated culture of transformed colonies and plates were incubated for 3–5 days at 25 °C or 37 °C. (**D**) ATPase assay was performed with PfRUVBL3 protein in presence of 100 ng of ssDNA (MP13mp18) or dsDNA (pUC19). The mean value of three independent experiments performed in duplicate is graphically depicted with ±SD. (**E**) ATPase activity of PfRUVBL3 (Wild-type) and PfRUVBL3 (ΔID) protein was measured and mean value of three experiments performed in duplicate was used to generate graph with ±SD. (**F**) Structure of PfRUVBL3 (ΔID) protein showing domain I (1–119 and 293–359) in pink color while domain III (360–483) in red color.

To determine whether PfRUVBL3 protein possess true ATPase activity, we performed ATPase assays with wild-type and Walker A mutant (where, lysine was converted into alanine) of PfRUVBL3 protein and investigated the consequences of mutating walker A motif known to be critical for ATP binding and hydrolysis (Fig. 4A). ATPase assay result showed that only the wild-type PfRUVBL3 could hydrolyze the ATP and the rate of ATP hydrolysis increases with increasing concentration of PfRUVBL3 protein while Walker A mutant form of PfRUVBL3 failed to hydrolyze ATP under similar conditions (Fig. 4B).

As we have shown earlier that PfRUVBL3 protein can complement the function of yeast RUVBL2 protein *in vivo*, we next wanted to determine whether ATPase activity of PfRUVBL3 protein is crucial for its function. We examined this by performing yeast complementation assay with wild-type and Walker A mutant form of PfRUVBL3. The result showed that only wild-type protein could rescue the growth of yeast cells at restrictive temperature while ATPase defective Walker A mutant of PfRUVBL3 failed to do so suggesting essential role of ATPase activity of PfRUVBL3 for executing its functions (Fig. 4C). Western blot analysis from pRS314-PfRUVBL3 (wild-type) and pRS314-PfRUVBL3 (Walker A mutant) transformed yeast cells showed expression level of recombinant proteins (Supplementary Fig. 3). RUVBL proteins are known to be partner of many chromatin remodeling complexes and transcription factors and needs access to chromatin. In order to investigate whether PfRUVBL3 ATPase activity is sensitive to the presence of DNA, we performed ATPase assay in presence of single-stranded DNA (ssDNA) and double-stranded DNA (dsDNA). Result showed that presence of dsDNA significantly enhanced the ATPase activity of PfRUVBL3 protein while under similar conditions there was no significant effect observed in presence of ssDNA (Fig. 4D). Insertion domain is unique to RUVBL proteins and we wanted to investigate its role in regulating the biochemical activities of PfRUVBL3, if any. ATPase assay performed with wild-type and PfRUVBL3 (ΔID) protein lacking insertion domain showed that deletion protein had significantly higher ATPase activity compared to the wild-type protein indicating a regulatory role of insertion domain in ATPase activity (Fig. 4E). Coomassie gel showed purified recombinant His-PfRUVBL3 (wild-type), PfRUVBL3 (Walker A mutant) and His-PfRUVBL3 (ΔID) protein (Supplementary Fig. 4). Structural analysis showed a normal conformation of insertion domain deleted PfRUVBL3 protein (Fig. 4F).

**Figure 5:**
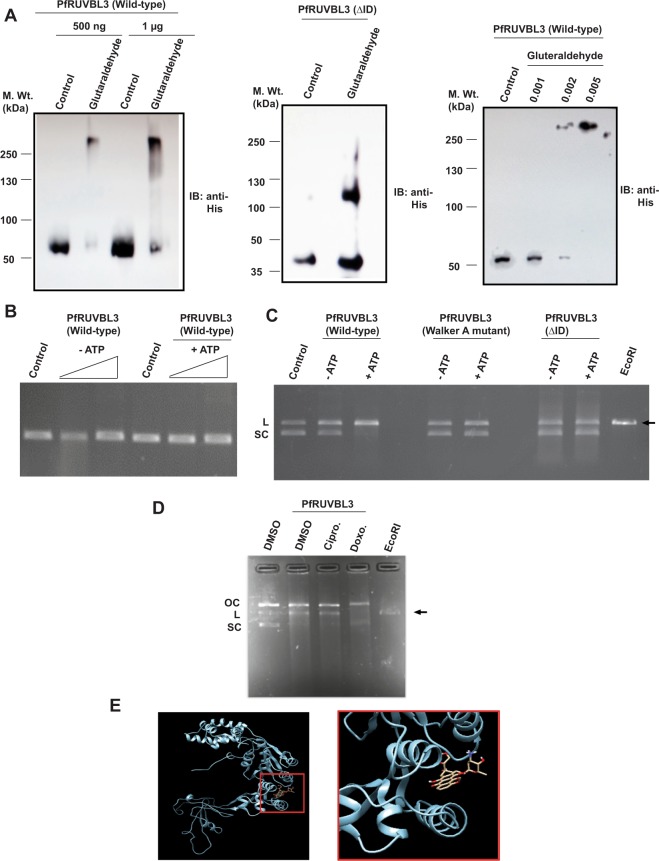
Oligomerization and DNA cleavage activity of PfRUVBL3 protein. (**A**) Oligomerization assay was performed with PfRUVBL3 (Wild-type) and PfRUVBL3 (ΔID) proteins in presence of 0.002% glutaraldehyde or as indicated with PfRUVBL3 (Wild-type). Proteins were resolved on 10% SDS-PAGE followed by Western blotting with anti-His antibody. (**B**) DNA cleavage assay was performed with His-PfRUVBL3 protein in presence of ssDNA (M13mp18) with or without ATP. Agarose gel was run to detect any change in migration of DNA. (**C**) Similarly, DNA cleavage assay was performed with His-PfRUVBL3 (Wild-type), His-PfRUVBL3 (Walker A mutant) or His-PfRUVBL3 (ΔID) proteins and dsDNA (pUC19) in presence or absence of ATP. Reaction samples were resolved on agarose gel to observe changes in DNA forms. *EcoRI* digested pUC19 was used as marker to identify linearized form respectively. (**D**) DNA cleavage assay was performed with PfRUVBL3 (Wild-type), pUC19 DNA and ciprofloxacin (10 µM) or doxorubicin (10 µM). SC, L and OC represents supercoiled, linear and open circular forms of DNA. (**E**) Docking study was performed with PfRUVBL3 protein and doxorubicin. Figure showed binding of doxorubicin with PfRUVBL3 protein at domain I (56–77 amino acids) and domain II (298–342 amino acids).

Since the members of AAA+ family of ATPases are known to form oligomeric structures (hexamers or dodecamers) as reported in other systems such as yeast and humans, we were interested to determine whether PfRUVBL3 existed as monomers or in oligomeric status. Besides, it is also important to determine the oligomeric status of PfRUVBL3 protein to understand its specific mechanism of action. We performed chemical crosslinking experiments with purified wild-type PfRUVBL3 and insertion domain deleted mutant of PfRUVBL3, using glutaraldehyde as the crosslinking reagent. The result showed that PfRUVBL3 protein form high molecular weight oligomers of approximate size of hexamer however under similar conditions PfRUVBL3 (ΔID) protein could only form trimers (Fig. 5A).

In few studies, insertion domain of RUVBL proteins has been shown to bind with DNA/RNA/protein, therefore to determine the binding specificity of PfRUVBL3 protein, we performed DNA binding assay with purified His-PfRUVBL3 protein using ssDNA or dsDNA. Although we could not detect any DNA binding activity of PfRUVBL3 protein in presence of ssDNA though, we observed that instead of binding to dsDNA, PfRUVBL3 protein cleaved dsDNA and converted the supercoiled DNA into linearized form. In addition, we observed that the DNA cleavage activity of PfRUVBL3 was dependent on its ATPase activity as well as on its insertion domain as either the absence of ATP or the insertion domain, failed to cleave the dsDNA under similar conditions (Fig. 5B,C). Furthermore, we found that the DNA cleavage activity exhibited by malaria parasite RUVBL3 protein could be similar to DNA cleavage activity shown by type II topoisomerases, which includes prokaryotic gyrase and eukaryotic topoisomerase II. We found that the treatment of PfRUVBL3 with doxorubicin (an inhibitor of eukaryotic type II topoisomerase) drastically inhibited its DNA cleavage activity, however, ciprofloxacin (an inhibitor of prokaryotic gyrase) failed to show any inhibition under similar conditions, indicating that the PfRUVBL3 DNA cleavage activity is similar to eukaryotic type II topoisomerases (Fig. 5D). *In silico* docking result showed that doxorubicin binds with both domains I and II of PfRUVBL3 (Fig. 5E). Together these results show that PfRUVBL3 protein has ATPase, oligomerization and a novel ATPase-dependent dsDNA cleavage activity that can be inhibited by eukaryotic type II topoisomerase inhibitors.

### Cloning, expression, purification of HAT protein PfMYST and generation of polyclonal antibody

**Figure 6:**
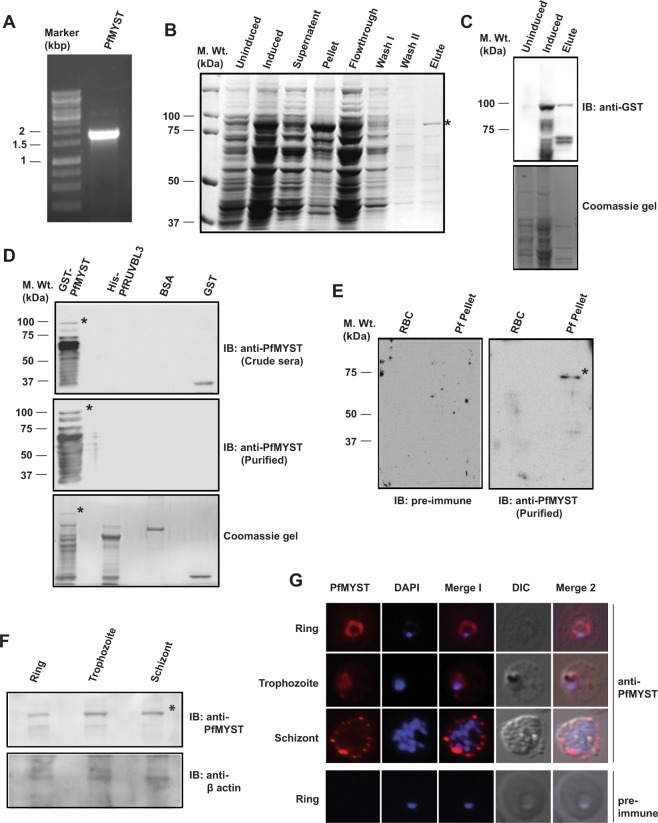
Cloning, recombinant protein purification, antibody generation and localization studies of PfMYST. (**A**) PCR amplification of PfMYST ORF. Agarose gel showed amplification of ~1.8 kbp ORF of PfMYST. (**B**) Protein expression and purification of recombinant PfMYST. BL21 DE3 (codon plus) cells transformed with pGEX6p2-PfMYST plasmid were cultured till ~1 OD and then induced with 0.5 mM IPTG for 8 hours at 37 °C. GST-PfMYST protein was purified by native purification method using glutathione sepharose beads. Coomassie gel showed purified protein expression profile and (*) showed the full-length band of GST-PfMYST protein at expected size of ~98 kDa. (**C**) Western blot by anti-GST antibody showed expression of GST-PfMYST protein in induced and elute lanes. (**D**) Characterization of generated anti-PfMYST antibody. Western blot was performed using crude anti-PfMYST immunized sera and purified anti-PfMYST antibody with purified recombinant proteins as indicated. (*) indicates full-length band of GST-PfMYST. (**E**) Endogenous expression of PfMYST protein. RBC and mixed stage parasite lysate were resolved on SDS-PAGE followed by Western blotting with purified anti-PfMYST antibody or pre-immune. Only PfMYST antibody detected single band at expected size of ~72 kDa. (*) Shows the band of PfMYST. (**F**) Stage-specific expression of PfMYST protein. Western blot analysis were performed with synchronized parasite lysates using purified anti-PfMYST and anti-β actin antibody. *Shows full length main band. Full length immunoblots are presented in Supplementary Fig. 7. (**G**) Intracellular localization of PfMYST. Immunofluorescence assay was performed with fixed smears from different stages of parasites as described in experimental procedures. Red signal shows localization of PfMYST while blue signal depicts DAPI stained nuclei.

In eukaryotic systems, RUVBL proteins have been shown to be partner of many chromatin-remodeling complexes like TIP60, Ino80, Swr1 and are essential either for complex formation or their activities. PfMYST, a homolog of human TIP60 is shown to be essential for parasite survival^7^. In humans, TIP60 is a complex of almost 18 proteins out of which RUVBL1/2 are essential for its activity^14^, however in malaria parasite no interacting partner of PfMYST has been identified till now. To purify PfMYST recombinant protein and to generate antibody, full-length ORF was amplified and cloned into pGEX6p2 vector and recombinant protein was purified under native conditions using affinity chromatography (Fig. 6A,B). Western blot analysis using anti-GST antibody showed specificity of the purified recombinant protein (Fig. 6C). GST-tagged PfMYST recombinant protein was purified at an expected size of ~98 kDa under native condition (Fig. 6B). GST-PfMYST was used as antigen to generate polyclonal antibody in mice and generated antibody was tested for specificity for PfMYST. Result showed that immunized crude sera recognized both GST-MYST and GST proteins, however purified sera against GST protein cross reacted only with GST-PfMYST protein (Fig. 6D). Western blot analysis was performed to detect the endogenous expression of PfMYST protein using mixed stage parasite lysate. Result showed single band at expected size of ~72 kDa in parasite lane only showing that the generated anti-PfMYST antibody can specifically recognize endogenous PfMYST protein (Fig. 6E). Under similar conditions, pre-immune sera did not show any cross reaction. Western blot with stage- specific parasite lysates showed the expression of PfMYST in all the stages of the developmental cycle (Fig. 6F). Generated graph showed that the expression of PfMYST is comparatively higher in trophozoite and schizont stages (Supplementary Fig. 5).

Next, to determine the intracellular localization of PfMYST, indirect immunofluorescence assay was performed using purified anti-PfMYST antibody in different stages of the parasite. Result showed that PfMYST is localized into parasitophorus vacuole in ring stage similar to PfRUVBL3 protein, however in trophozoite stage it showed distribution majorly in the nucleus. In schizont stage, PfMYST localized predominantly on periphery of the infected erythrocyte (Fig. 6G). Under similar conditions pre-immune sera failed to show any signal. Our results are in contrast to previous study that showed PfMYST to be localized majorly in the nucleus in all the stages^7^. The reason of the difference could be that in the previous study GFP-tagged protein was used for localization that might have affected its actual intracellular distribution. Overall, these results show that PfMYST is expressed in all the stages of the developmental cycle and displays dynamic intracellular localization.

### PfRUVBL3 colocalizes and interacts with PfMYST during intra-erythrocytic developmental cycle stages

**Figure 7:**
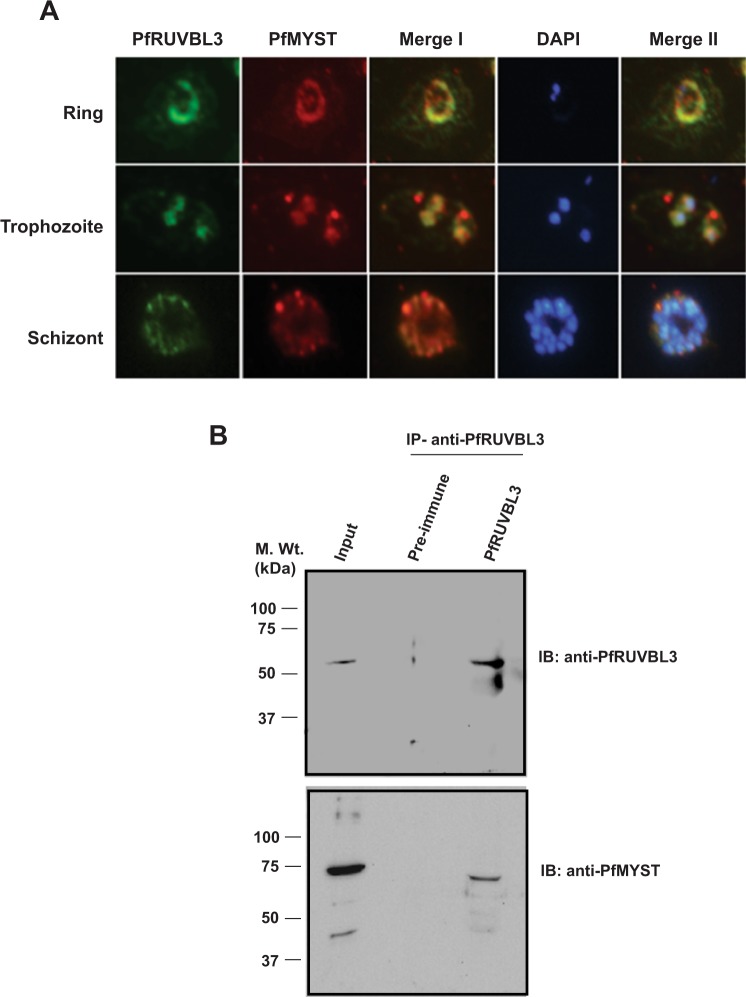
Colocalization and interaction of PfRUVBL3 and PfMYST. (**A**) Immunofluorescence assay was performed with fixed smears of parasite from different stages of IDC using anti-PfRUVBL3 (rabbit origin) and anti-PfMYST (mice origin) antibody to detect their intracellular localization. Green signal in the figure depicts PfRUVBL3 and red signal shows PfMYST localization. DAPI stained (blue color) the nucleus. (**B**) Co-immunoprecipitation assay was performed with mixed stage parasite culture using PfRUVBL3 antibody or pre-immune sera. Immunoprecipitated lysate was resolved on SDS-PAGE followed by Western blotting with anti-PfRUVBL3 or anti-PfMYST antibody. Light chain specific secondary antibody (rabbit and mice from Merck Milipore) were used in these Western blot analysis.

In human and yeast, RUVBL proteins are partners of TIP60 complex and we were interested to determine if in malaria parasite, PfRUVBL3 is an interacting partner of PfMYST protein. Colocalization study in different stages of IDC showed that both PfRUVBL3 and PfMYST perfectly colocalized in parasitophorus vacuole during ring stage (Fig. 7A). As the parasite progressed to trophozoite stage PfRUVBL3 got concentrated mainly in the nucleus and PfMYST also showed colocalized nuclear staining. In the schizont stage, both the proteins were found to be localized on the periphery and showed significant overlapping punctate pattern (Fig. 7A). Next, to determine whether both these proteins physically interact with each other, immunoprecipitation (IP) assay was performed with the mixed stage parasite culture. PfRUVBL3 protein was pulled down using anti-PfRUVBL3 antibody or pre-immune sera followed by Western blotting. Western blot result showed that PfRUVBL3 protein band was detected only in anti-PfRUVBL3 antibody IP lane while there was no signal in pre-immune IP lane showing successful pull down of PfRUVBL3 protein from the parasite lysate (Fig. 7B). Western blot of same samples with purified anti-PfMYST antibody showed presence of PfMYST protein in input and PfRUVBL3 IP lane only (Fig. 7B). This shows that PfRUVBL3 and PfMYST interact with each other in the parasite.

### PfMYST colocalizes with H3K9me1 histone in parasitophorous vacuole during ring stage

**Figure 8:**
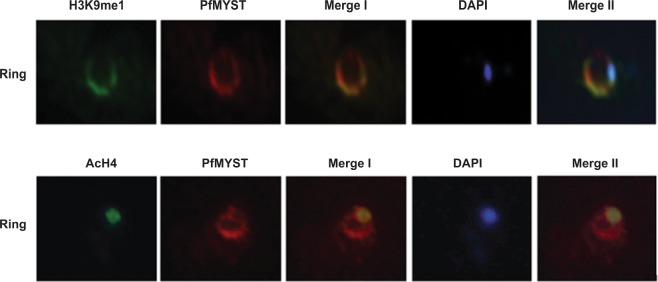
Colocalization of PfMYST and H3K9me1 histone. Colocalization studies were performed by immunofluorescence assay in ring stage of parasite. Red color shows localization of PfMYST while green color depicts localization of H3K9me1 histone and AcH4 respectively. Blue color depict DAPI stained nucleus.

Plasmodium has conserved histones like humans and a previous study has shown dynamic localization of histones in various stages of its development^15^. Histone 3 with monomethylated modification at lysine 9 (H3K9me1) has been shown to exclusively localize in the parasitophorous vacuole during the ring stage. Since PfMYST also exhibited similar localization pattern, we performed colocalization studies of PfMYST with H3K9me1 in various stages. Interestingly, we observed that PfMYST and H3K9me1 showed clear colocalization in the parasitophorus vacuole only in the ring stage (Fig. 8A). However, in other stages H3K9me1 exhibited dispersed signal in cytosol and nucleus and did not colocalize significantly with PfMYST (data not shown). For comparison, when we performed similar colocalization experiment of PfMYST with acetylated histone 4 (AcH4), we failed to observe PfMYST colocalization with AcH4 in the ring stage and both the proteins showed distinct distribution pattern where PfMYST was localized in the parasitophorous vacuole while AcH4 was localized exclusively in the nucleus (Fig. 8A). Together, these results show that PfMYST and H3K9me1 histone specifically colocalize with each other in ring stage of developmental cycle.

## Discussion

RUVBL proteins (RUVBL1 & 2) are known to regulate diverse cellular processes and have been shown to be essential for survival in *S*. *cerevisiae*, drosophila and *C*. *elegans*^16–19^. Interestingly, *Plasmodium* possess three putative homologs of RUVBL in comparison to its eukaryotic counterparts, however their biological significance remains mostly speculative till date. Therefore, to expand our knowledge about the basic biology of this deadly parasite and to determine the biological implication of RUVBL proteins in Plasmodium, we need to functionally characterize them in detail. In the present study, we have shown that PfRUVBL3 protein is a true homolog of yeast RUVBL2. Since in yeast, RUVBL proteins are shown to be highly essential for survival and are known to regulate transcription of almost 5% of its genes^16^, the ability of PfRUVBL3 to strongly complement the functions of yeast RUVBL2 suggests conserved or comparable functions for this protein. In addition, we found that PfRUVBL3 ATPase activity is indispensable for yeast survival, as the walker A mutant of PfRUVBL3 failed to support yeast growth under heat stress. A common feature of AAA+ family member proteins is the formation of higher order oligomers (mainly hexamers) and recent studies on RUVBL proteins structure of yeast and human have shown that these proteins predominantly form hexameric structure along with dodecameric population^20,21^. In our study, we have shown that purified PfRUVBL3 protein can also form similar high molecular weight oligomers of hexamer size, however it remains to be determined whether all the three parasite RUVBL proteins interact and make oligomers to function in sync with other eukaryotic systems.

Using further biochemical analysis, we showed that PfRUVBL3 possess DNA cleavage activity by which it can convert supercoiled dsDNA into linearized form and thus may alter DNA topology in a similar manner as eukaryotic type II topoisomerases do. Interestingly, ATP-dependent DNA cleavage activity of PfRUVBL3 was found to be reliant on its insertion domain. Insertion domain (domain II) is unique to RUVBL proteins and has been speculated to interact with DNA/RNA. In addition, RUVBL proteins are also known to associate with multi-protein chromatin remodeling complexes and transcription factors that often need to alter the targeted local chromatin architecture in order to perform their functions. Hence, it is reasonable to speculate that PfRUVBL proteins with intrinsic DNA cleavage activity can assist opening of supercoiled DNA and restructure chromatin architecture to facilitate either loading of the complex, ease the accessibility of targeted DNA or promote histone modification/exchange in a context-dependent manner. However, it would be interesting to explore the preferred site of DNA cleaved by PfRUVBL3 protein and the catalytic mechanism involved in this event. Additionally, our data showed that insertion domain also act as a modulator of PfRUVBL3 ATPase and oligomerization activities. Deletion of PfRUVBL3 insertion domain remarkably enhanced its ATPase activity. Furthermore, PfRUVBL3 protein lacking insertion domain can only make trimer in contrast to human and yeast homologs that can make dodecamer even in absence of insertion domain.

Further, in an effort to identify interacting partner of PfRUVBL3 protein, we investigated its interaction with the most likely partner, PfMYST protein, a homolog of TIP60 protein in *Plasmodium*. RUVBLs are also known to exist as partner proteins of TIP60 complex in human and yeast^14^. We observed that both PfRUVBL3 and PfMYST constitutively expresses in all the stages of IDC starting from ring to schizont and colocalize with each other. Interestingly, the colocalization of both the proteins in ring stage-parasitophorous vacuole merge with H3K9me1 histone. Studies on human genome have shown that dynamic alteration in chromatin organization of actively transcribed genes by exchange of histones, facilitate access of transcriptional machinery and H3K9me1 mark is associated with active promoters and is found to be enriched at transcription start sites (TSS)^22^. In addition, TIP60 is also known to interact with methylated histones through its chromodomain and thus act as code reader for its interaction with chromatin. For instance, during DNA damage TIP60 gets localized at double-stranded breaks (DSBs) by binding to H3K9me3 histones through its chromodomain^23^. In presence of estrogen, TIP60 binds to Estrogen Receptor α (ERα) targeted promoters by binding to H3K4me1 histone through its chromodomain^24^. Another study has shown that TIP60 chromodomain prefers binding to methylated and acetylated peptides^25^. Based on these evidences, it might be possible that PfMYST in a complex with RUVBL3 protein might interact with H3K9me1 histones through its chromodomain and incorporate it to targeted gene promoters followed by acetylation of nearby histones to poise them for transcriptional activation during ring and trophozoite stages. In this event dsDNA cleavage activity of PfRUVBL3 might help in deposition or exchange of histones at the targeted gene promoters to regulate transcription,

## Methods

### Parasite culture

Human malaria parasite *P*. *falciparum* (3D7 strain) was cultured in human erythrocytes in RPMI 1640 medium (containing 0.2% NaHCO_3_, 0.5% albumax, 10 µg/ml gentamicin sulfate and 50 µg/ml hypoxanthine) at 37 °C and mixture gas environment (90% nitrogen, 5% carbon dioxide and 5% oxygen). Routine cultures were maintained at 5% hematocrit and parasite stages and parasitemia were determined by microscopic examination of Giemsa-stained *Plasmodium*-infected blood smears. For synchronization of the cultures, parasites were treated with 5% sorbitol in ring stage and verified by Geimsa staining.

### Primers and DNA manipulations

All the primers used in the study, were synthesized and purchased from IDT (Supplementary Table 1). PfRUVBL3 gene was amplified by PCR from *P*. *falciparum* 3D7 strain genomic DNA as template using specific primers. Approximately 1450 bp PCR product was obtained for PfRUVBL3. Amplified PCR product was cloned into pET28a vector under *SacI* and *XhoI* restriction sites to generate pET28a-PfRUVBL3 clone. Similarly, to generate pGEX6p2-PfMYST construct, PfMYST was amplified and cloned into pGEX6p2 vector under *BamHI* and *EcoRI* restriction sites. Point mutants or deletion mutants of RUVBL3 were generated by overlapping PCR method using mutation specific primers (Supplementary Table 1) using wild-type clone as template.

For yeast complementation assays, PfRUVBL3 ORF was cloned into pRS416 yeast expression vector under *BamHI* and *EcoRI* sites under galactose promoter. Subsequently, the cassette containing the galactose promoter, PfRUVBL3 ORF and terminator region, was excised out by using *SacI* and *KpnI* restriction enzymes and then ligated into another yeast vector pRS314 vector, containing tryptophan as a selection marker. To generate ScRUVBL2 clone, ORF was amplified by PCR using *Saccharomyces cerevisiae* genomic DNA as template and specific primers (Supplementary Table 1). Amplified ORF was initially cloned into pRS416 under *BamHI* and *EcoRI* site and subsequently into pRS314 vector using similar strategy as mentioned earlier. All the generated constructs were confirmed by sequencing.

### Recombinant protein purification and antibody generation

To purify tagged-recombinant proteins from bacteria, protocol described elsewhere was followed with modifications^26^. *E*. *Coli* BL21 DE3 cells were transformed with indicated plasmids and were cultured at 37 °C till it reached 0.4–0.6 optical density. 1 mM of isopropyl-β-thiogalactopyranoside (IPTG) was added to cultures to induce production of recombinant proteins. After 6 hrs, cells were harvested and lysed in ice-cold lysis buffer (1X PBS, 2 mM EDTA, 5 mM DTT, 1% Triton X-100, 10% glycerol, 0.5 mM PMSF and 1X protease inhibitor cocktail) followed by addition of lysozyme (10 mg/ml) and lysate was incubated at 4 °C for 30 minutes. Lysate was sonicated thrice at 4 °C and followed by centrifugation at 14,000 rpm for 30 minutes to separate the clear lysate. Equilibrated Ni-nitrilotriacetic acid (Ni-NTA) beads were added to the lysate and mixture was continuously rotated at 4 °C for 1 hr. Protein-bound Ni-NTA beads were separated by centrifugation and washed with wash buffer (1X PBS containing 20 mM imidazole) thrice and bound proteins were eluted by elution buffer (1X PBS containing 1 M imidazole). Eluted proteins were dialyzed in dialysis buffer (50 mM Tris.Cl pH- 8, 2 mM EDTA, 100 mM NaCl, 10% glycerol, 0.2 mM PMSF) and aliquots were prepared and stored at −80 °C. For purification of GST-tagged proteins similar protocol was followed except that glutathione agarose beads were used to trap recombinant proteins and elution was performed by elution buffer containing 10 mM glutathione at 4 °C for 30 minutes.

Purified recombinant His-PfRUVBL3 and GST-PfMYST proteins were used as antigen to generate polyclonal antibody in rabbit and mice respectively from Abgenex India Pvt company, Bhubaneshwar. Generated antibodies were tested for specificity by Western blotting against purified recombinant proteins and parasite lysate.

### Immunofluorescence assay and Western blot analysis

For immunofluorescence assay, thin smears of *P*. *falciparum* infected erythrocytes from cultures of various stages were prepared on glass slides. Cells were fixed in chilled methanol for 10 minutes. The fixed parasites were permeabilized using 0.1% Triton-X 100 in 1X phosphate-buffered saline (PBS) (v/v) for 15 minutes at room temperature followed by blocking with BSA. Slides were incubated either with PfRUVBL3, PfMYST, H3K9me1 (Merck Millipore) or AcH4 (Merck Millipore) antibody or their corresponding pre-immune sera at the dilution of 1:250 at 4 °C for 8–10 hours in a humidified chamber followed by washing with 1X PBS three times. Slides were then incubated with secondary antibody (Alexa Flour 488 anti-rabbit IgG or Alexa Flour 596 anti-mice IgG) for an hour at 1:2000 dilution at room temperature in a humidified chamber followed by washing with 1X PBS. Air dried slides were counterstained with antifade mount containing DAPI (Thermo) to stain the nucleus. Parasites were visualized under fluorescence microscope (Nikon Ti Eclipse) for the detection of signals.

Western blotting using standard protocol was performed to examine the expression of PfRUVBL3 protein. Pellets of synchronized parasite cultures were treated with 0.05% saponin for 5 minutes at room temperature, washed with 1X PBS followed by lysis in lysis buffer (50 mM Tris. Cl (pH- 8.0), 150 mM NaCl, 1 mM DTT, 5 mM EDTA, 0.5% NP-40, and 1X protease inhibitor cocktail). Equivalent amount of protein samples derived from distinct stages were resolved by SDS-PAGE and then transferred onto the PVDF membrane. Blots were probed with indicated either PfRUVBL3, PfMYST or anti-β actin (Genscript, catalog number A00702) followed by incubation with corresponding secondary antibody. To visualize the protein band, blots were treated with ECL reagent (BioRad) and signal was developed using FluorChem system (Protein simple).

### Yeast complementation assay

Yeast RUVBL2 temperature-sensitive strain (YRVB2a) was a kind gift from Prof. Anindya Dutta (University of Virginia, USA). Yeast strain was transformed either with pRS314, pRS314-ScRUVBL2 or pRS314-PfRUVBL3 constructs using lithium chloride method. Transformed cells were plated onto tryptophan-dropout media and incubated at 25 °C for 4–5 days. To examine the complementation efficiency of the transformed cells, master plate was prepared by streaking transformed colonies on tryptophan drop out plate and clones were selected for complementation study by making the replica of transformed cells and culturing them at permissive temperature (25 °C) or restrictive temperature (37 °C).

### ATPase, oligomerization and DNA cleavage assay

ATPase assay was performed as described somewhere else with modifications^27^. Fresh malachite green reagent was prepared by adding malachite green (0.0812%, w/v), polyvinyl alcohol (2.32%, w/v), ammonium molybdate (5.72%, w/v, in 6 M HCl) and distilled water in the ratio 2:1:1:2 and kept at room temperature for 2 hours. Purified proteins (His-PfRUVBL3 wild-type and mutant proteins) were added to the ATPase buffer (100 mM Tris pH-8.0, 20 mM MgCl_2_, 2 mM ATP) and incubated at 30 °C for 1 hour and subsequently malachite green solution was added to the reaction mixture and further incubated for 20 minutes at room temperature. Samples were diluted 1:15 ratio in distilled water followed by measurement of absorbance at 620 nM.

Glutaraldehyde cross-linking assay was performed to detect the oligomerization activity of PfRUVBL3 proteins. Purified proteins were incubated with or without 0.002% glutaraldehyde in 20 µl reaction buffer at 37 °C for 30 minutes. The reaction was terminated by adding 2X Lamelli buffer and samples were boiled at 95 °C for 10 minutes. Samples were then resolved by SDS-PAGE followed by Western blotting using anti-His antibody.

For determining DNA cleavage activity, purified recombinant proteins were incubated with single-stranded (MP13mp18) or double-stranded (pUC19) DNA in reaction buffer (20 mM Tris–HCl Ph-8.0, 1 mM MgCl_2_, 100 mM KCl, 8 mM dithiothreitol, 4% sucrose and/or 5 mM of 1 M ATP solution) at 30 °C for 1 hour. Reaction was terminated by adding 6X loading dye and samples were resolved in agarose gel. *EcoRI* digested samples were simultaneously run as control.

### Immunoprecipitation assay

For immunoprecipitation assay, parasite-infected erythrocytes were harvested and treated with 0.05% saponin. Parasites were then lysed in lysis buffer (50 mM Tris-Cl pH- 8.0, 150 mM NaCl, 2 mM EDTA, 0.5% Triton X-100, 0.1% SDS and 1X protease inhibitor cocktail) for 40 minutes at 4 °C with continuous rotation followed by sonication. Subsequently, the samples were incubated overnight with anti-PfRUVBL3 antibody or pre-immune sera at 4 °C followed by incubation with equilibrated protein A sepharose beads (Thermo Scientific) for 2 hrs. Protein-bound beads were washed thrice with lysis buffer and bound proteins were resolved by SDS-PAGE followed by Western blotting with anti-PfRUVBL3 or PfMYST antibody.

### Structural and docking analysis

Structure of PfRUVBL3 was modeled using homology modeling with SWISS–MODEL software^28^ by taking human Rvb1/Rvb2 heterohexamer in INO80 complex (PDB ID: 5OAF) as template with 68% identity with the target sequence. Human RUVBL2 structure was also modeled using same template of human Rvb1/Rvb2 heterohexamer in INO80 complex (PDB ID: 5OAF) with 100% identity. Superimposition of modeled PfRUVBL3 structure with the structure of the hRuvBL2 resulted in rmsd of 0.17 Å. Similarly, structure of insertion domain deletion mutant of PfRUVBL3 (ΔID) was modelled using template of dodecameric human RuvBL1:RuvBL2 complex with truncated domains II (PDB ID: 2XSZ) structure. For docking analysis, ligand doxorubicin was downloaded from ZINC database in mol2 format and converted to pdb using OpenBabel GUI version 2.4.1. AutoDock Tools version 1.5.6 was used to convert pdb to pdbqt format. AutoDock Vina version 1.1.2 (The Scripps Research Institute) was used to predict best binding pocket for doxorubicin to PfRUVBL3 protein. UCSF Chimera software version 1.12 was used for visualization and analysis of the results obtained from AutoDock Vina^29^.

## Electronic supplementary material

Supplementary Information
