# Supplementary material for: Plasmodium falciparum RUVBL3 protein: a novel DNA modifying enzyme and an interacting partner of essential HAT protein MYST
Source: Sci Rep. 2018 Jul 19;8:10917. doi: 10.1038/s41598-018-29137-8 (PMC6053374; doi:10.1038/s41598-018-29137-8)
Supplement: Supplementary file 1 — Supplementary Information [file 41598_2018_29137_MOESM1_ESM.docx]

Title- ***Plasmodium falciparum* RUVBL3 protein: a novel DNA modifying enzyme and an interacting partner of essential HAT protein MYST**

Authors- **Utsav Sen, Himani Saxena, Juhi Khurana, Akshaykumar Nayak, Ashish Gupta^*^**

Affiliations**- Department of Life Sciences, Shiv Nadar University, Greater Noida, India 201314**

Running title- ***Functional characterization of PfRUVBL3 protein***

^*^Corresponding author:

**Ashish Gupta**

**Assistant Professor**

**Department of Life Sciences**

**Shiv Nadar University**

**Greater Noida, India-201314**

Email- **ag315@snu.edu.in**

Tel.- **07838523796**

**Keywords*:*** Plasmodium, malaria, parasite, protein purification, chromatin remodeling, RUVBLs, MYST

**Supplementary Figure 1**


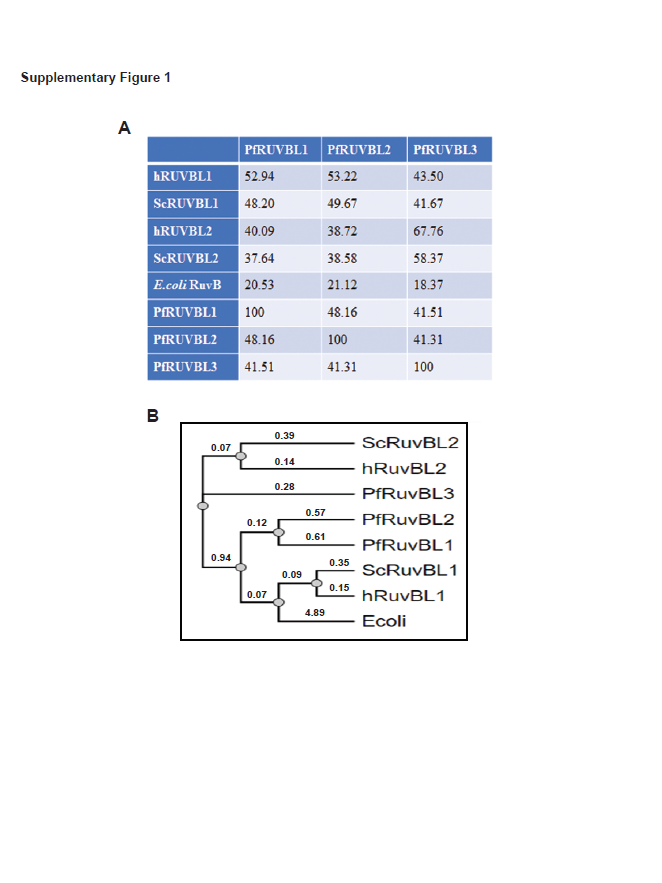


**Supplementary Figure 1:** **Sequence comparison and phylogenetic analysis of RUVBL proteins.** **(A)** Table shows percentage identity of Plasmodium RUVBL proteins with human, *Saccharomyces cerevisiae* and *E. coli* homologs. PfRUVBL3 showed highest identity with human RUVBL2 protein. **(B)** Phylogenetic tree was generated using PhyML 3.0 software and depict the relationship between RUVBL proteins from different organisms as indicated in the figure.

**Supplementary Figure 2**


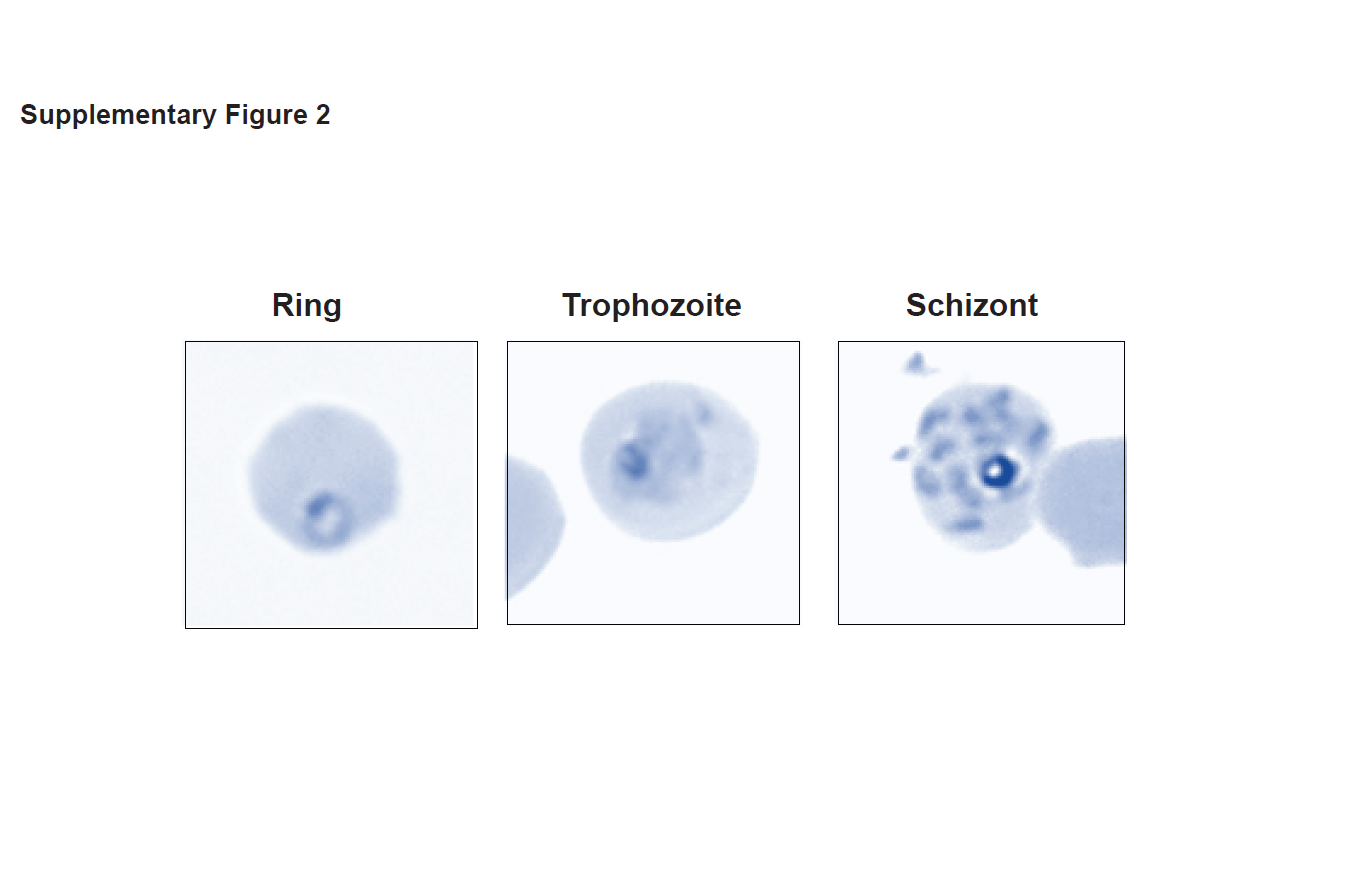


**Supplementary Figure 2:** Geimsa-stained stages of asexual intra-erythrocytic developmental cycle of *Plasmodium falciparum*.

**Supplementary Figure 3**


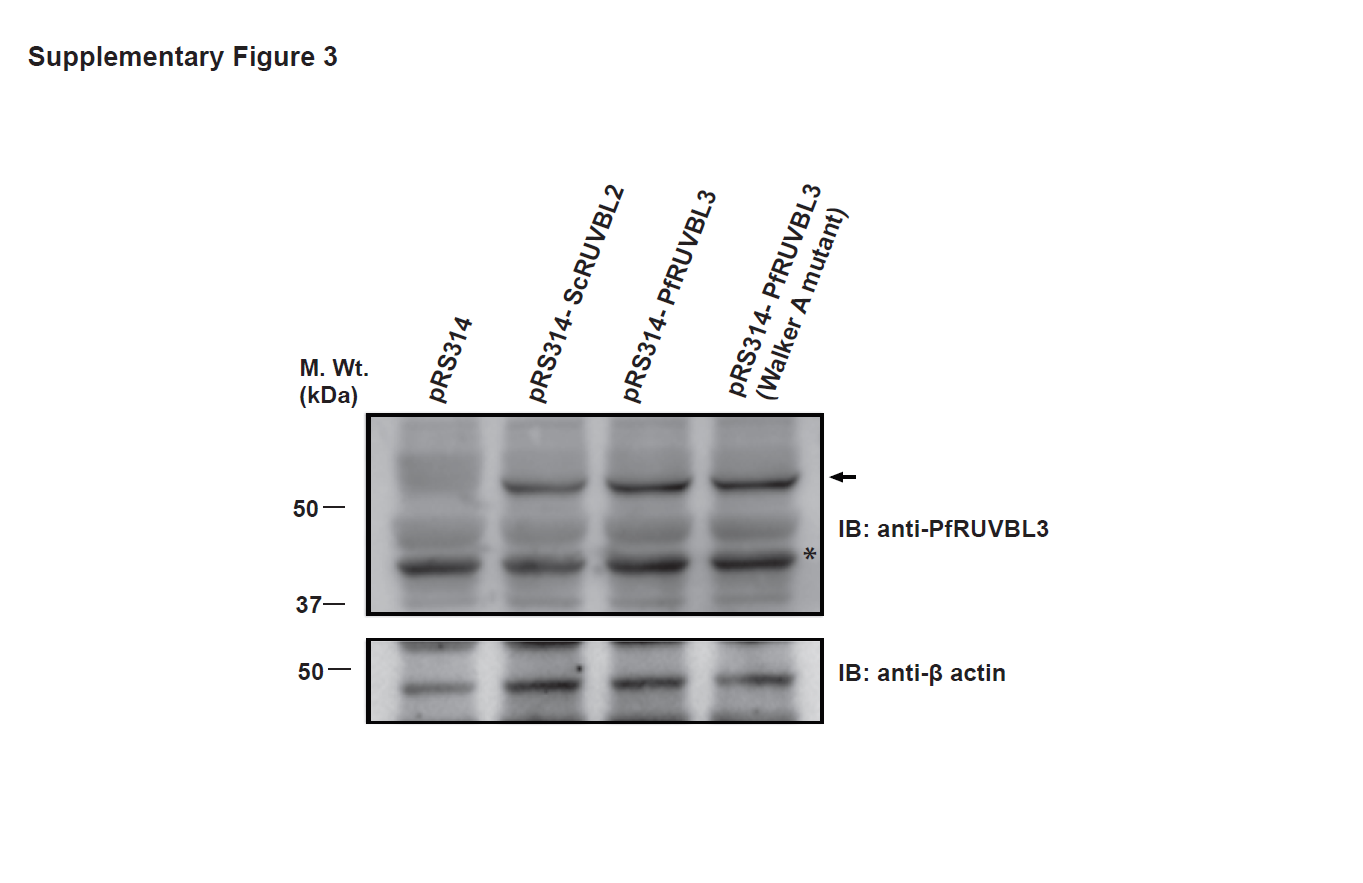


**Supplementary Figure 3:** **Expression of recombinant proteins from transformed yeast strain.** Yeast temperature sensitive (ts) strain was transformed with indicated plasmids and transformed cells were cultured at 25^o^C. Equal amount of lysates was resolved on SDS-PAGE and Western blot analysis was performed with anti-PfRUVBL3 antibody or anti-β actin antibody. Arrow shows the band of recombinant proteins at their expected size. (*) shows a cross reacting band.


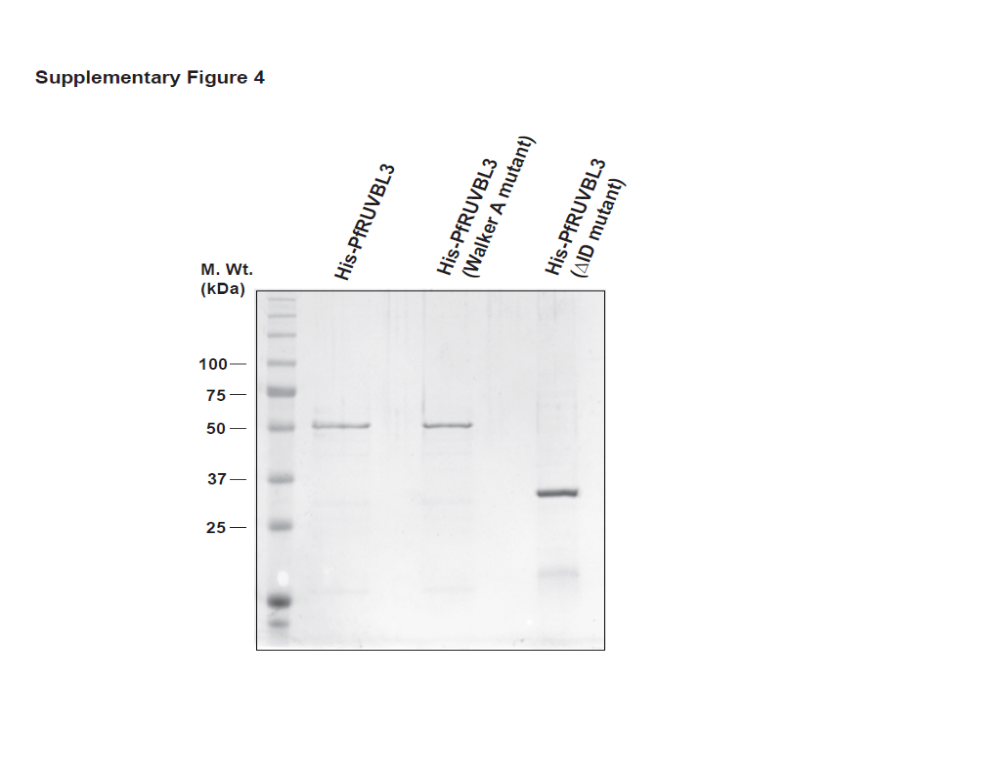
**Supplementary Figure 4**

**Supplementary Figure 4:** **Recombinant proteins purified from bacteria**. *E.coli* BL21 DE3 (codon plus) cells were transformed with pET28a-PfRUVBL3 (Wild-type), pET28a-PfRUVBL3 (Walker A mutant) or pET28a-PfRUVBL3 (ΔID) plasmids. Recombinant proteins were purified and coomassie gel shows the purified recombinant proteins.

**Supplementary Figure 5**


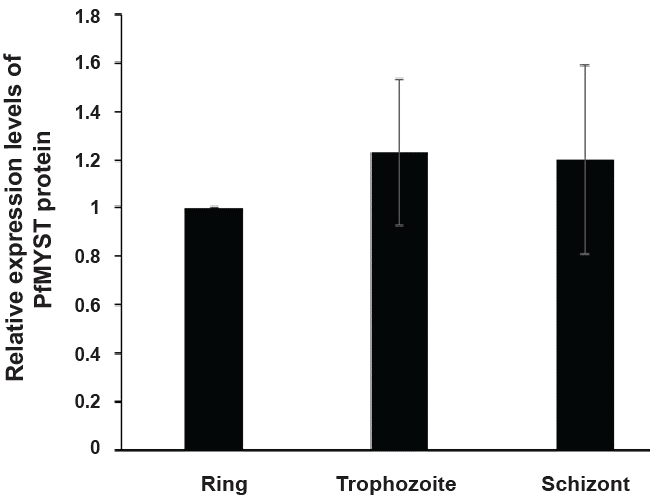


**Supplementary Figure 5:** Graph depicts the normalized values of stage-specific expression of PfMYST as shown in Figure 6F. Value of ring stage was taken as 1.

**Supplementary Figure 6**


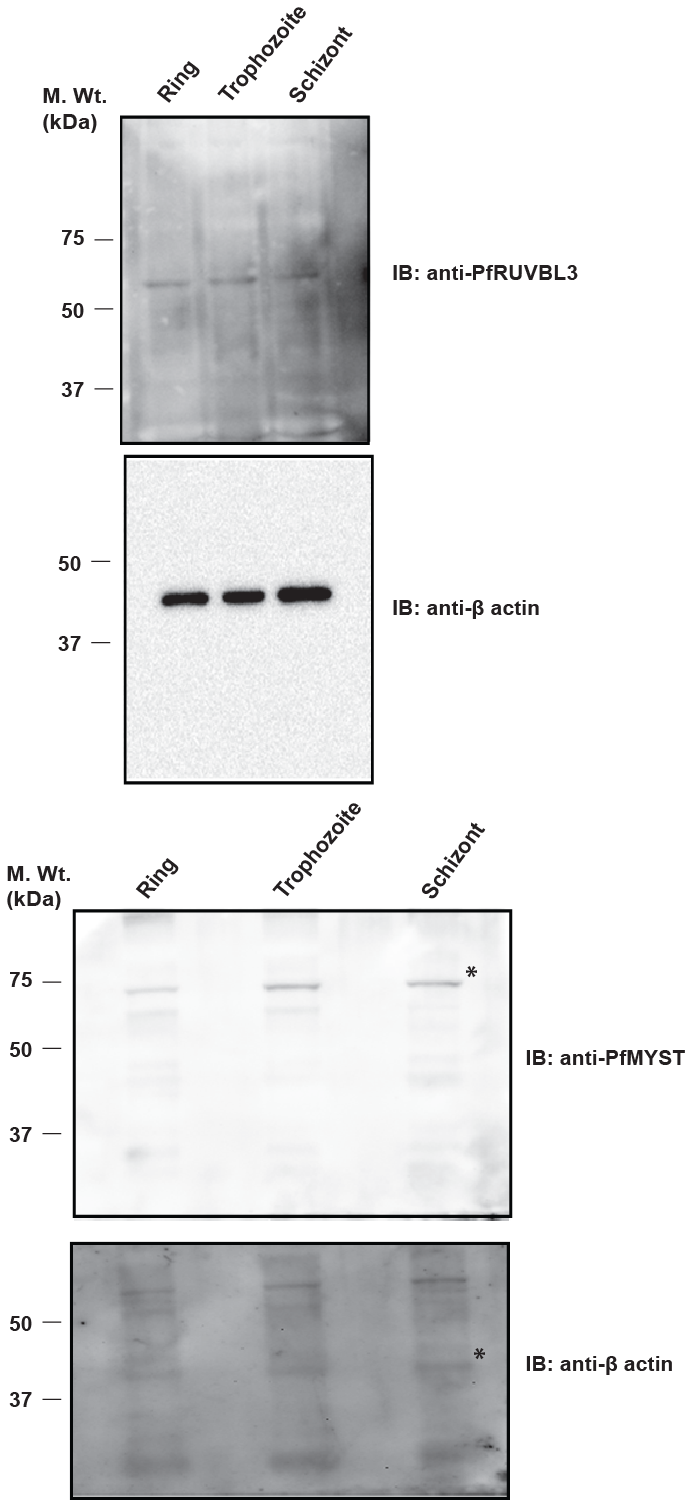


**Supplementary Figure 6:** Full-length immunoblots showing expression of indicated proteins. (*) shows main protein band.

**Supplementary Table S 1**

List of primers used in the study.

| **Primers** | **Primer Sequence (5’ – 3’)** |
| --- | --- |
| *Pf*RVB3FLFwSacI | ACCGAGCTCATGAAGCTCGAAGAAGTGAAAG |
| *Pf*RVB3FLRvXhoI | CCGCTCGAGTTAATTACTTGTACTTGAATTTTCC |
| *Pf*RVB3WAMutFwd | CAACCAGGTACAGGGGCAACAGCCATTGCT |
| *Pf*RVB3WAMutRev | CATAGCAATGGCTGTTGCCCCTGTACCTGG |
| *Pf*RVB3IDdelFwd | CAAGCTTTTAGATCTGTCTTATTTATTGAT |
| *Pf*RVB3IDdelRev | TTCATCAATAAATAAGACAGATCTTCTAAAAGCTTG |
| *Pf*RVB3FLFwClaI | ACCATCGATATGAAGCTCGAAGAAGTGAAAG |
| *Pf*RVB3FLRvSalI | CCGGTCGACTTAATTACTTGTACTTGAATTTTCC |
| *Pf*MYSTFLFwBamHI | CCGGATTCATGGTCCTGGGGGGAAAGA |
| *Pf*MYSTFLRvEcoRI | CCGAATTCTTATTCATATAAGGCCAAATAATAATCA |
| ScRVB2FLFwBamHI | CGGGATCCATGTCGATTCAAACTAGTGATCC |
| ScRVB2FLRvEcoRI | CGGAATTCTTATTCCGTAGTATCCATGGCA |
